# WW domain-binding protein 2 overexpression prevents diet-induced liver steatosis and insulin resistance through AMPKβ1

**DOI:** 10.1038/s41419-021-03536-8

**Published:** 2021-03-03

**Authors:** Zhe Zheng, Yue Li, Siyuan Fan, Jie An, Xi Luo, Minglu Liang, Feng Zhu, Kai Huang

**Affiliations:** 1 Clinic Center of Human Gene Research, Union Hospital, Tongji Medical College, Huazhong University of Science and Technology, Wuhan, China; 2 Department of Cardiology, Union Hospital, Tongji Medical College, Huazhong University of Science and Technology, Wuhan, China; 3 Department of Cardiology, Handan First Hospital, Handan, China

**Keywords:** Lipid signalling, Non-alcoholic fatty liver disease

## Abstract

Nonalcoholic fatty liver disease (NAFLD) is prevalent clinically and can lead to more serious chronic liver disease. However, the pathological mechanism is still unclear, and thus, there are no approved drugs on the market. Transcriptional coactivator WW domain-binding protein 2 (WBP2) is a newly discovered oncogene that has an important relationship with the occurrence and development of breast cancer and mediates the interaction between Wnt and various other signaling pathways. The expression level of WBP2 was decreased in NAFLD. Overexpression of WBP2 with AAV in vivo alleviated liver fat deposition and insulin resistance induced by a high-fat diet (HFD). Knockdown of WBP2 with AAV aggravated HFD-induced fatty liver and insulin resistance. In vitro experiments showed that in the human normal hepatocyte cell line LO2 and primary hepatocytes isolated from mice, overexpression of WBP2 reduced fat deposition, and knocking out or knocking down WBP2 aggravated PA-induced fat deposition. Through mass spectrometry, we found that WBP2 can bind to AMPKβ1, and by mutating AMPKβ1, we found that WBP2 can induce phosphorylation of AMPKβ1 at S108 and then activate the AMPK pathway to affect lipid metabolism. The effect of WBP2 on NAFLD provides a possible new direction for future research on NAFLD.

## Introduction

Globally, nonalcoholic fatty liver disease (NAFLD), a metabolic disease, has been increasing. In NAFLD, many lipid droplets rich in triglycerides are deposited in hepatocytes in the liver in the absence of excessive alcohol consumption^1,2^. As the disease progresses, NAFLD progresses from simple steatosis to nonalcoholic steatohepatitis, leading to fibrosis and necrosis of the liver^3,4^. Current research results indicate that NAFLD’s most important pathophysiological factors include a series of metabolic risk factors, such as insulin resistance (IR), dyslipidemia, and obesity. Even so, at this stage, there are no effective means for control and treatment. Some researchers suggest that the regulation of individual key molecules or signaling pathways might be our future direction.

Adenosine monophosphate-activated protein kinase (AMPK) is a cellular energy-sensing enzyme that regulates the metabolic state. One catalytic subunit, α, and two regulatory subunits, β and γ, make up the AMPK complex^5,6^. When cells are in an energy stress state that could increase the AMP/ATP ratio, AMP binds to the γ subunit and allosterically activates this enzyme^7,8^. Thus, energy homeostasis is restored by AMPK activation, which could inhibit energy-consuming anabolic processes and regulate catabolic processes^5,9,10^. The AMPK complex is mainly activated by phosphorylation of Thr172 in the activation loop of subunit α. Liver kinase B1, Ca^2+^/calmodulin-dependent protein kinase kinase β, and transforming growth factor β kinase have been found to phosphorylate the Thr172 site^11^. There are two regulatory subunits of the AMPK complex, and the β subunit can be phosphorylated; phosphorylation at the Ser108 site was related to the activity of catalytic subunit α, phosphorylation at the Ser182 site and nuclear translocation of the catalytic subunit^12^. AMPK could have a major regulatory effect on various systemic metabolic pathways^13^. Therefore, the study of AMPK activation and regulation has become an important research direction for treating metabolic diseases.

WW domain-binding protein 2 (WBP2) protein can bind to proteins containing the WW domain. The WW domain is rich in proline motif structures, which can regulate protein–protein interactions^14^. In breast cancer, WBP2 can combine with YAP and act as a bridge connecting the Hippo and Wnt/β-catenin pathways^15–17^. In addition, WBP2 can be used as an adaptor protein to bind to pax8 and play a crucial role in the differentiation of thyroid cells^18^. In addition, reports have proven that WBP2 can act as a transcriptional coactivator of the estrogen receptor^16,19,20^. Clinical studies have found that patients with increased WBP2 expression in breast cancer patients have a relatively poor prognosis^17^. Existing research has shown that WBP2 has a function in Wnt/β-catenin signaling, tissue growth, and cancer.

While current evidence has shown that WBP2 has diverse biological functions, the effect of WBP2 on NAFLD is still unknown. Our research showed that WBP2 was downregulated in the liver of a mouse model of NAFLD; overexpression of WBP2 in the mouse liver inhibited steatohepatitis and IR caused by a high-fat diet (HFD). The effect of WBP2 on NAFLD relies on AMPKβ1 Ser108 phosphorylation, which could enhance AMPK complex activity.

## Materials and methods

### Animals

Six-week-old C57BL/6J mice were housed in a specified pathogen-free animal room with a 12-h light/12-h dark cycle and free access to food and water. For diet-induced NAFLD, 6-week-old male C57BL/6J mice were randomized into two groups: one group was fed a HFD (60.9% fat, 21.8% carbohydrate, and 18.3% protein; H10060) for 16 weeks, and the other group was fed a normal chow diet (NCD) (4% fat, 78% carbohydrate, and 18% protein; H10010, HFK Bioscience, Beijing, China). Gene-edited *ob/ob* mice provided by HFK Bioscience (Beijing, China) were fed an NCD for 8 weeks. The mice were sacrificed at the time point, blood samples were collected for testing, and liver tissue was collected for fixation with paraformaldehyde or storage with frozen liquid nitrogen. All experimental procedures in this study were approved by the Animal Use Subcommittee of Tongji Medical College of Huazhong University of Science and Technology.

### Cell culture

The HEK293T and normal human hepatocyte cell line LO2 were provided by Procell Biotech (Wuhan, China). The cells were cultured in DMEM containing 10% fetal bovine serum and 1% penicillin-streptomycin. The protocol for isolation, purification, and culture of primary hepatocytes from mice was described previously^21^. Briefly, mice were anesthetized by intraperitoneal injection of 60 mg/kg body weight sodium pentobarbital. The abdominal cavity was opened to expose the abdomen and find the mouse liver portal vein, 5 ml of liver perfusion solution (D-Hank’s, 10 mM HEPES, and 0.5 mM EGTA) was infused to remove blood. Then the infusion was continued with digestive buffer (D-Hank’s,10 mM HEPES, and 5 mM collagen II) at 2.5–5 ml per minute until the liver lost its elasticity and the color changed. Then, the surface membrane of the liver was removed, it was cut into pieces and filtered with a strainer, and the hepatocytes were resuspended in DMEM with 10% serum. The viable cells were counted with Trypan blue staining, and then the hepatocytes were planted on a fibronectin-coated cell culture plate. Palmitate (PA) (0.25 mM; P0500; Sigma-Aldrich, St. Louis, MO, USA) was used to establish an in vitro cell lipid deposition model. Cells were stained with 1 µg/ml Nile red (IN0170; Solarbio, China) staining solution for 10 min to assess lipid deposition in the cells. Cellular Nile red-stained lipids were observed using fluorescence microscopy and quantitated with ImageJ software. A triglyceride colorimetric assay kit (10010303; Cayman) was used to detect intracellular triglyceride levels. All these cells were cultured under 37 °C and 5% CO_2_.

### Western blot

Whole-cell lysates were isolated from tissues or cells at the indicated times using RIPA lysis buffer with PhosSTOP phosphatase inhibitor (Roche Diagnostics, Barcelona, Spain) and complete protease inhibitor cocktail (Roche). The BCA protein assay kit (Thermo Scientific, Waltham, MA, USA) was applied to determine protein concentrations. Equal amounts of protein were subjected to PAGE; after that, proteins were transferred to a PVDF membrane (Millipore, Billerica, MA, USA). After incubation in nonfat milk in TBST to block the membranes, the membranes were incubated with the following primary antibodies: anti-WBP2 (Proteintech, 12030-1-AP), anti-β-actin (Abcam, ab8226), anti-AMPKα (Proteintech, 66536-1-Ig), anti-AMPKβ1 (Cell Signaling Technology, 12063), anti-AMPKγ1 (Proteintech, 10290-1-AP), anti-GST (Proteintech, 66001-2-Ig), anti-p-AMPKα (Thr172) (Cell Signaling Technology, 50081), anti-ACC (Cell Signaling Technology, 3676), anti-p-ACC (Cell Signaling Technology, 3661), anti-insulin receptor substrate 1 (IRS1) (Cell Signaling Technology, 2382), anti-AKT (Cell Signaling Technology, 4691), anti-glycogen synthase kinase 3β (GSK3β) (Cell Signaling Technology, 9315), anti-p-IRS1 (Millipore, 09-432), anti-p-AKT (Cell Signaling Technology, 4060), anti-p-GSK3β (Cell Signaling Technology, 9322), anti-p-AMPKβ1 (Ser108) (Affinity Biosciences Cat# AF8238, RRID: AB_2840300), anti-p-AMPKβ1 (Ser182) (Affinity Biosciences Cat# AF8030, RRID:AB_2840093), anti-Flag (Proteintech, 66008-3-Ig), and anti-anti-phospho-Ser/Thr (Abcam, ab117253) at 4 °C overnight. Then, the samples were incubated with the horseradish peroxidase-conjugated secondary antibody for 1 h at room temperature. Chemiluminescence signals were detected by the ChemiDoc XRS+ imaging system (Bio-Rad, Hercules, CA, USA).

### Immunofluorescence

For analysis of the colocalization of WBP2 and AMPKβ1 in hepatocytes, LO2 cells were seeded on coverslips in 6-well plates. The cells were fixed in 10% formalin and then blocked with 2.5% goat serum. The mouse WBP2 antibody (Proteintech; 66585-1-Ig) and the rabbit AMPKβ1 antibody (Proteintech; 10308-1-AP) were used as primary antibodies. Anti-mouse IgG (H+L), F(ab’)2 Fragment (Alexa Fluor^®^ 488 Conjugate) (Cell Signaling Technology; 4408) and Anti-rabbit IgG (H+L), F(ab’)2 Fragment (Alexa Fluor^®^ 594 Conjugate) (Cell Signaling Technology; 8889) antibodies were applied as the corresponding secondary antibodies. WBP2 expression and localization in the liver sections of mice were investigated using WBP2 and albumin (ALB, a marker of hepatocytes) co-staining. The mouse ALB-specific antibody (Proteintech, 66051-1-Ig) and the rabbit WBP2 antibody (Proteintech; 12030-1-AP) were used as primary antibodies, and anti-rabbit IgG (H+L), F(ab’)2 Fragment (Alexa Fluor^®^ 488 Conjugate) (Cell Signaling Technology; 4412) and Anti-mouse IgG (H+L), F(ab’)2 Fragment (Alexa Fluor^®^ 594 Conjugate) (Cell Signaling Technology; 8890) antibodies were applied as the corresponding secondary antibodies. Cell nuclei were stained with DAPI. Immunofluorescence images were obtained using a fluorescence microscope (Olympus).

### Recombinant adenovirus and adeno-associated virus production

Recombinant adenovirus containing WBP2, WBP2 shRNA (a short hairpin RNA targeting WBP2) (mouse WBP2 shRNA1: CCGGGAGATTAAGCAGCCGGTGTTTCTCGAGAAACACCGGCTGCTTAATCTCTTTTTTG, mouse WBP2 shRNA2: CCGGCTACTGTTTCTGGGAATTATTCTCGAGAATAATTCCCAGAAACAGTAGTTTTTG), green fluorescent protein (GFP, as a negative control), or scramble shRNA (as a nonspecific control) was applied to infect primary hepatocytes in vitro.

The recombinant adeno-associated virus (AAV) system (type 8), which contained control and WBP2 with a liver-specific promoter (ALB promoter) or scramble shRNA and WBP2 shRNA, was employed to manipulate the expression of WBP2 in vivo via tail vein injection. Mice were injected via the tail vein with 100 μl of virus containing 2 × 10^11^ VG of the AAV8 vector genomes.

Recombinant adenovirus containing AMPKβ1 and GFP (as a negative control) was employed to manipulate the expression of AMPKβ1 in vivo via tail vein injection. Purified adenovirus with an ALB-specific promoter was injected into the livers of the mice through tail vein injection (100 μl per mouse with the adenovirus containing 5 × 10^10^ pfu/ml). All adenoviruses and AAVs were provided by Obio Technology Corp, Ltd. (Shanghai, China).

### SiRNA transfection

The human normal hepatocyte cell line LO2 was seeded in plates and cultured to 80% confluence. The cells were then transfected with AMPKβ1 small interfering RNA (siRNA) targeting the 3’-UTR of AMPKβ1 or control scramble siRNA (Santa Cruz Biotechnology, Dallas, TX, USA) using Lipofectamine LTX transfection reagent (Thermo Fisher Scientific) according to the manufacturer’s instructions. The validated siRNA sequence was as follows: GCCTGGCTATGGAACTAAATA. The medium was replaced with a fresh medium 6 h after transfection. The cells were cultured for 24 h before treatment with the indicated agents.

### Mouse experiments

Body weight and blood glucose levels were measured at the corresponding time points. After 16 weeks of high-fat feeding, glucose tolerance tests (GTTs) and insulin tolerance tests (ITTs) were performed. For the GTTs, the mice were fasted for 6 h and injected intraperitoneally with 1 g/kg glucose. For the IR experiment, the mice were fasted for 6 h and intraperitoneally injected with 0.75 U/kg of insulin. After injection for 15, 30, 60, and 120 min, the blood glucose concentration was measured using a blood glucometer to collect tail vein blood from the mouse. For the acute tissue insulin signaling tests, overnight-fasted male mice after 16 weeks on an HFD were anesthetized with the muscular injection of ketamine/xylazine, followed by portal vein injection of human insulin (0.5 U/kg body weight) or vehicle saline. Five minutes later, the livers were collected to determine phosphorylation of IRS1, total IRS1, phosphorylation of AKT, total AKT phosphorylation of GSK3β, and total GSK3β by western blotting.

### Histological analysis

Paraffin sections were stained with hematoxylin and eosin (H&E) to observe the changes in lipid accumulation. Oil red O staining was performed on frozen liver sections to assess the liver’s accumulation of lipid droplets. Photographs were taken by an optical microscope (Olympus, Tokyo, Japan).

### Quantitative real-time PCR

Total RNA was extracted from mouse cells and liver tissues using TRIzol (D9108A, TaKaRa Bio). Reverse transcription of isolated RNA into complementary DNA was performed using the RR037A PrimeScript™ RT reagent Kit (Perfect Real Time) (TaKaRa). SYBR Green (Vazyme) was used to quantify the amplification products on a PRISM 7900 Sequence Detector System (Applied Biosystems, Foster City). All genes were quantified using β-actin as an internal control. Primer sequences are available upon request.

### Mouse hepatic lipid analyses and serum assays

The triglycerides and nonesterified fatty acids (NEFAs) were measured using a commercial kit (290-63701 for the triglyceride assay and 294-63601 for the NEFA assay; Wako, Osaka, Japan). An ADVIA 2400 chemical system analyzer (Siemens, Tarrytown, NY, USA) was used to determine the serum alanine aminotransferase (ALT) and aspartate aminotransferase (AST) concentrations according to the instructions to evaluate liver function.

### CRISPR/Cas9 knockout (KO) cell line

CRISPR-Cas9 technology was used to knock out WBP2 in the human normal hepatocyte cell line LO2, according to the protocol described by Zhang’s lab^22^. Briefly, the second exon of WBP2 was chosen as a target to submit to the online CRISPR design website (http://www.rgenome.net/), and then, the designed sgRNA was cloned into px459 (Addgene #62988). The sgRNA sequence of WBP2 was 5′-TGTGAGTTCCACGTGATCAT-3′. The sgRNA was delivered using the pX459 expression plasmid, which contains both the Cas9 nuclease and a puromycin resistance gene. The constructed plasmid was transfected into LO2 cells and selected with puromycin 2 days after transfection. A total of 1 × 10^5^ cells were divided into six 96-well plates to obtain as many single clones as possible. Western blot and PCR were used to identify positive single clones.

### Immunoprecipitation

The cells were lysed in ice-cold IP buffer (20 mM Tris-HCl, pH 7.4, 150 mM NaCl, 1 mM EDTA, 1% Triton X-100, 10 μg/ml aprotinin, 10 μg/ml leupeptin, 0.5 mM β-glycerophosphate disodium salt hydrate and 1 mM phenylmethylsulfonyl) containing complete protease inhibitor (no. 04693132001, Roche) and centrifuged at 12,000 g for 15 min. The cell lysate was collected and incubated with protein G Sepharose beads (no. 11719416001, Roche) with the indicated antibody overnight at 4 °C and then washed in immunoprecipitation buffer. The immune complexes were collected and immunized with the indicated antibody.

### GST precipitation assay

A GST precipitation assay was used to determine the direct interaction between WBP2 and AMPKβ1. Rosetta (DE3) *Escherichia coli* cells were transfected with the plasmid pGEX-4T-1-GST-WBP2, pGEX-4T-1-GST-AMPKα, pGEX-4T-1-GST-AMPKβ1, or pGEX-4T-1-GST-AMPKγ1. The cells were then treated with 0.5 mM thiogalactoside (IPTG) (no. 15529019, Thermo Scientific) to achieve an optical density (OD600) of 0.8 at 600 nm. *E. coli* cells were lysed, and the extract was mixed with glutathione-Sepharose 4B beads (no. 17075601, GE Healthcare Biosciences, Buckinghamshire, UK) for 2 h at 4 °C. Then, the protein-loaded beads were incubated with purified Flag-tagged AMPKα, Flag-tagged AMPKβ1, Flag-tagged AMPKγ1, or Flag-tagged WBP2. The interacting proteins were eluted with elution buffer (50 mM Tris-HCl [pH 8.0] and 20 mM reduced glutathione) and immunoblotted with anti-Flag antibodies.

### AMPK ADP-GloTM kinase assays

WBP2 and AMPK complexes and the AMPKβ1 S108A mutation were purified as described above. AMPK complexes with wild-type (WT) AMPKβ1 or S108A mutant AMPKβ1 subunits, 0.2 µg/µl SAMStide (HY-p0136; MCE) and 150 μM ATP in kinase buffer (40 mM Tris, pH 7.5; 20 mM MgCl_2_; 0.1 mg/ml BSA; 50 µM DTT; 100 µM AMP) with or without WBP2 were incubated at room temperature for 60 min. The ADP-GloTM Kinase Assay (V9101; Promega) was applied to measure the activity of the AMPK complex. Five microliters of the mixtures above was added to 5 µl of ADP-GloTM Reagent and then incubated at room temperature for 40 min to remove ATP that had not been consumed. Ten microliters of Kinase Detection Reagent was added to newly synthesized ATP using the luciferin reaction and incubated at room temperature for 30 min. Then, the activity of AMPK kinase could be measured by reading the luminescence.

### Statistical analysis

Data represented the mean ± SEM and were compared between or among groups by two-tailed Student’s *t*-test or one-way ANOVA. *P* < 0.05 was considered statistically significant.

## Results

### Hepatic WBP2 expression was decreased in NAFLD

**Fig. 1:**
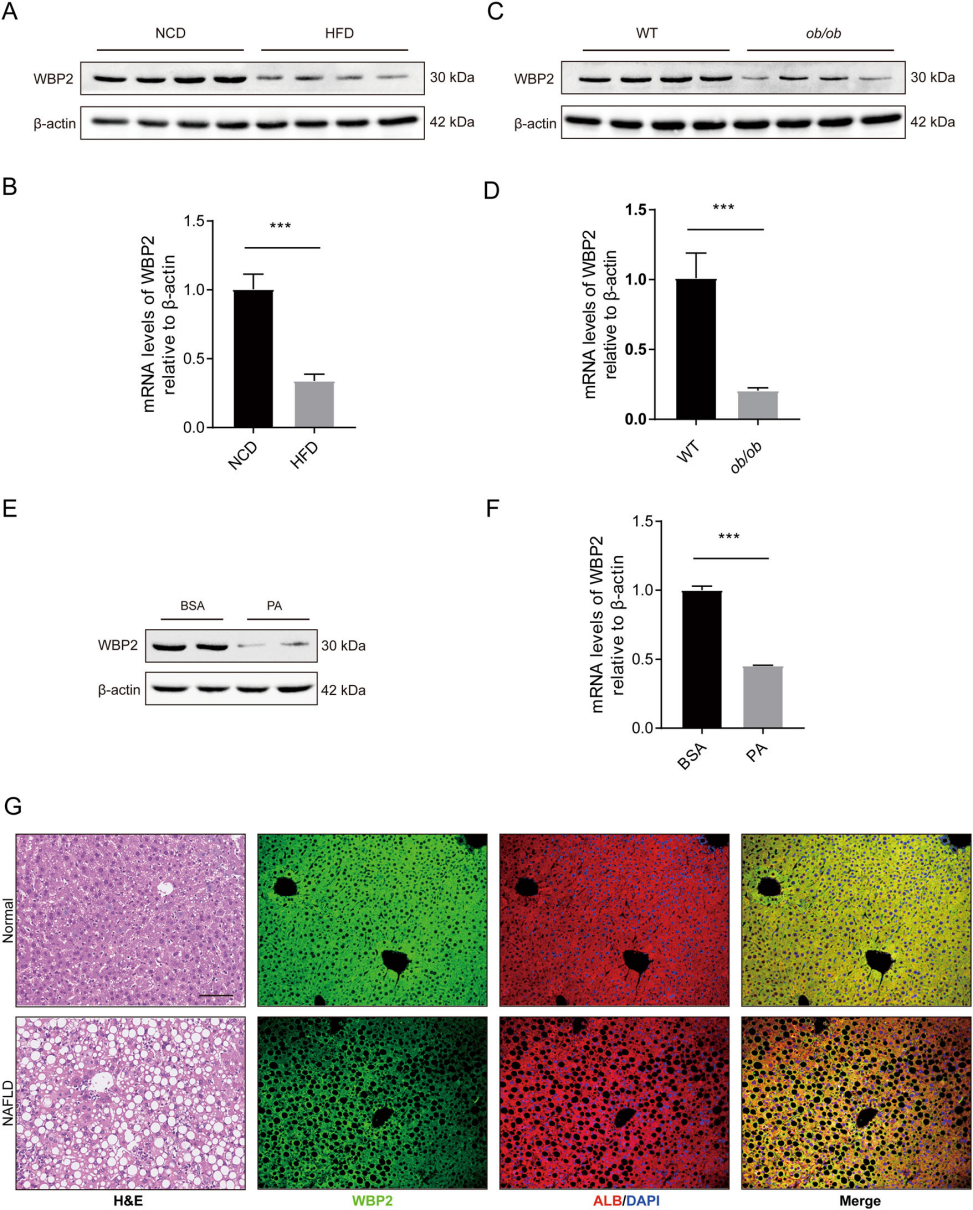
WBP2 is downregulated in livers with hepatic steatosis. **A**, **B** The representative protein (**A**) and mRNA (**B**) levels of WBP2 in the livers of the mice subjected to an HFD and an NCD for 16 weeks (*n* = 8/group). **C**, **D** Representative protein (**C**) and mRNA (**D**) levels of WBP2 in the livers of the WT or *ob/ob* mice (*n* = 8/group). **E**, **F** The primary hepatocytes isolated from the livers of wild-type mice was stimulated with PA (0.25 mM) for 24 h, and WBP2 expression was examined by western blots (**E**) and RT-PCR (**F**) (*n* = 3 independent experiments). The protein and mRNA expression levels were normalized to the β-actin levels. **G** Representative images of H&E staining and immunofluorescence staining for WBP2 (green), ALB (red) and DAPI (blue) in the liver samples from mice fed an HFD or an NCD for 16 weeks (*n* = 8/group). Scale bar, 100 μm. Data are expressed as the mean ± SEM, ***P < 0.001.

To determine whether WBP2 is involved in the occurrence and development of fatty liver, we tested the expression of WBP2 in the livers of the mice with NAFLD. In the livers of the mice fed an HFD, the expression of WBP2 at both the protein and mRNA levels was lower than that in the mice fed an NCD (Fig. 1A, B). In addition, compared with that of the normal mice, in the liver of the *ob/ob* mouse model of severe NAFLD, we found that the expression of WBP2 was decreased at the protein and mRNA levels (Fig. 1C, D). Moreover, we stimulated primary hepatocytes isolated from the livers of WT mice with PA. The protein and mRNA levels of WBP2 decreased (Fig. 1E, F). However, oleic acid stimulation of liver cells did not affect the expression of WBP2 (Supplementary Fig. S1A, B).

Mice were fed an HFD for 16 weeks, and H&E staining was performed on mouse liver sections. Obvious lipid droplet deposition was detected (Fig. 1G). To detect the location and expression of WBP2 in mouse fatty liver, we performed immunofluorescence co-staining with WBP2 antibody and ALB (hepatocyte marker) antibody (Fig. 1G). The results showed that WBP2 was mainly expressed in the cytoplasm of hepatocytes and that the expression of WBP2 was decreased in the liver of the HFD-fed mice.

### WBP2 could affect cellular lipid deposition caused by PA stimulation in vitro

**Fig. 2:**
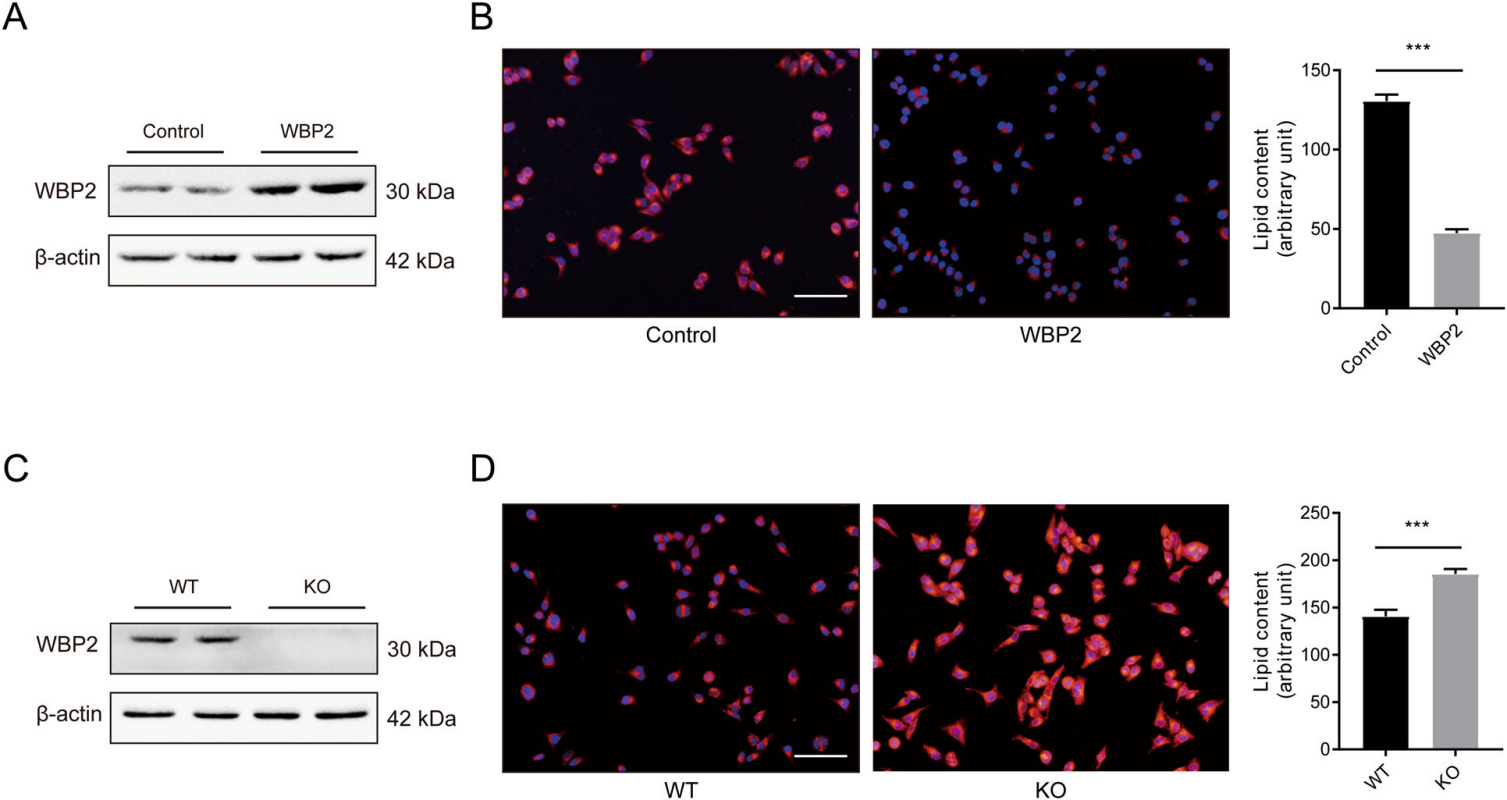
The effect of WBP2 on lipid deposition in primary hepatocytes stimulated by PA. **A**, **C** A WBP2-overexpressing cell line (**A**) and a WBP2 knockout cell line (**C**) were established in LO2 cells. The protein expression of WBP2 was tested by western blot analysis. **B** Representative photomicrographs with Nile red staining are shown for the control and WBP2-overexpressing cells exposed to PA (0.25 mM) for 12 h. **D** Representative photomicrographs with Nile red staining are shown for the WT and WBP2 KO cells exposed to PA (0.25 mM) for 12 h. The Nile red-stained area was quantified by ImageJ software. Scale bar, 100 μm. Data are expressed as the mean ± SEM, ***P < 0.001.

WBP2 decreased under the stimulation of fatty liver and PA, suggesting that WBP2 might have a role in lipid metabolism. For further investigation, the human liver cell line LO2 was transfected with the control plasmid or WBP2 plasmid, and the WBP2 KO cell line was constructed by the CRISPR Cas9 system. Western blot assays were applied to detect the WBP2 protein expression efficiency (Fig. 2A, C). Nile red staining was used to indicate the degree of lipid deposition in the cells. When the expression of WBP2 increased, the accumulation of intracellular lipid droplets induced by PA decreased, and when the level of WBP2 decreased, the accumulation of intracellular lipid droplets induced by PA increased compared with those of the control group (Fig. 2B, D). These results were further validated in primary hepatocytes isolated from adult mice (Supplementary Fig. S2).

### WBP2 overexpression reduced HFD-induced hepatic steatosis and insulin resistance

**Fig. 3:**
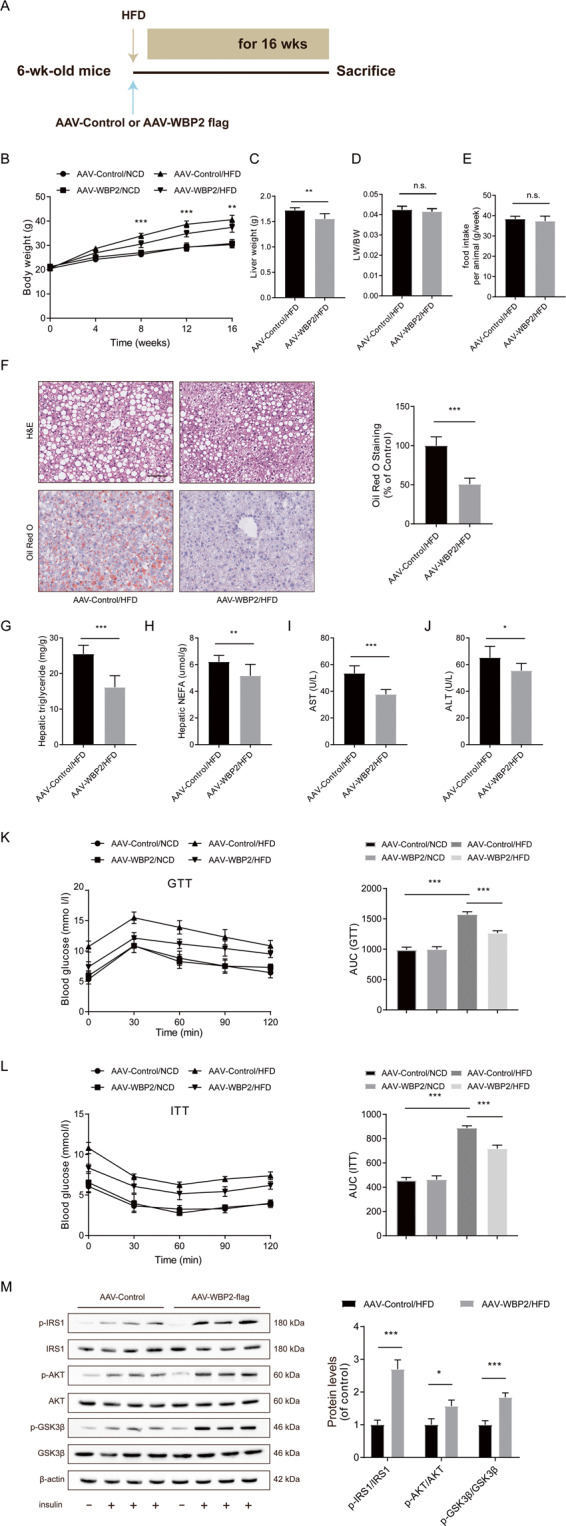
Overexpression of WBP2 alleviates HFD-induced hepatic steatosis and insulin resistance. **A** Schedule of WBP2 overexpression. Six-week-old mice were injected with AAV-Control (as a control) or AAV-WBP2 Flag (overexpressing WBP2) via the tail vein. **B** Body weight, **C** liver weight, **D** LW/BW, and **E** food intake of the AAV-Control mice and the AAV-WBP2 mice (*n* = 8/group). **F** Representative images of H&E and Oil red O staining of liver tissues from the AAV-Control mice and the AAV-WBP2 mice fed an HFD for 16 weeks. Scale bar, 100 μm. **G**, **H** Triglyceride, NEFA, and **I**, **J** ALT and AST levels in the livers of the AAV-Control mice and the AAV-WBP2 mice fed an HFD for 16 weeks (*n* = 8/group). **K**, **L** Intraperitoneal glucose tolerance tests (GTTs; 1 g/kg) (**K**) and intraperitoneal insulin tolerance tests (ITTs; 0.75 U/kg) (**L**) were performed on the AAV-Control mice and the AAV-WBP2 mice at the 16th week of food administration. The corresponding area under the curve (AUC) of the blood glucose level was calculated (*n* = 8/group). **M** Representative western blot analysis (*n* = 3 western blots for each band) of phosphorylated (p-) and total IRS1, AKT, and GSK3β expression in the livers of the AAV-Control mice and the AAV-WBP2 mice fed an HFD for 16 weeks that received insulin treatment (*n* = 2 mice in each group without insulin injection; *n* = 6 mice in each group with insulin injection). Data represent the mean ± SEM, *P < 0.05, **P < 0.01, ***P < 0.001 and n.s. indicates no significance between the two indicated groups.

To further clarify the effect of WBP2 on lipid metabolism in vivo, we injected hepatocyte-specific WBP2 AAV through the tail of C57BL/6J mice (Fig. 3A). To ensure that the expression of WBP2 was specifically expressed in the mouse liver for a long period of time, we tested the expression of WBP2 in the mouse liver at different time points after different injections. The western blot results showed that AAV-WBP2 could achieve long-term expression in the mouse liver (Supplementary Fig. S3A). After feeding the animals an HFD for 16 weeks, we tested the expression of exogenous WBP2 in different tissues (Supplementary Fig. S3B). AAV-WBP2 could achieve specific expression of WBP2 in the mouse liver due to the ALB-specific promoter. The immunofluorescence colocalization of WBP2 and ALB showed that WBP2 was mainly expressed in liver cells (Supplementary Fig. S3C).

After 16 weeks of high-fat feeding, compared to the negative-control AAV group, the AAV-WBP2 group had an obvious decrease in the body weight and liver weight (Fig. 3B, C); however, the liver-to-body weight and food intake showed no meaningful variation (Fig. 3D, E). In addition, regarding body weight, the difference between the two groups of mice fed an NCD was not significant (Fig. 3B). Moreover, the results of H&E and Oil red O staining showed that the AAV-WBP2 group had lower liver lipid deposition than the AAV-control group when fed an HFD for 16 weeks (Fig. 3F). The former group also had decreased liver triglycerides and NEFAs (Fig. 3G, H). In addition, WBP2 could prevent the abnormal liver function induced by HFD, and the plasma levels of AST and ALT were decreased in the overexpression group (Fig. 3I, J). To explore the effects of WBP2 on glucose metabolism and insulin sensitivity, we conducted GTTs and ITTs on the mice before they were sacrificed. The difference in the areas under the curve in GTTs and ITTs showed that we could increase glucose tolerance and insulin sensitivity by WBP2 overexpression (Fig. 3K, L).

Next, for the acute tissue insulin signaling tests, we tested the changes in vital molecules in the insulin signaling pathway in which phosphorylation was consistently increased in the liver tissue of the AAV-WBP2 mice. Insulin-induced phosphorylation of key factors in insulin signaling, such as IRS1, AKT, and GSK3β, was much higher in the livers of the AAV-WBP2 mice than in those of the AAV-control group after a 16-week HFD (Fig. 3M). These results indicated that overexpression of WBP2 could reduce fatty liver and IR caused by an HFD.

### Knockdown of WBP2 aggravated HFD-induced fatty liver and insulin resistance

**Fig. 4:**
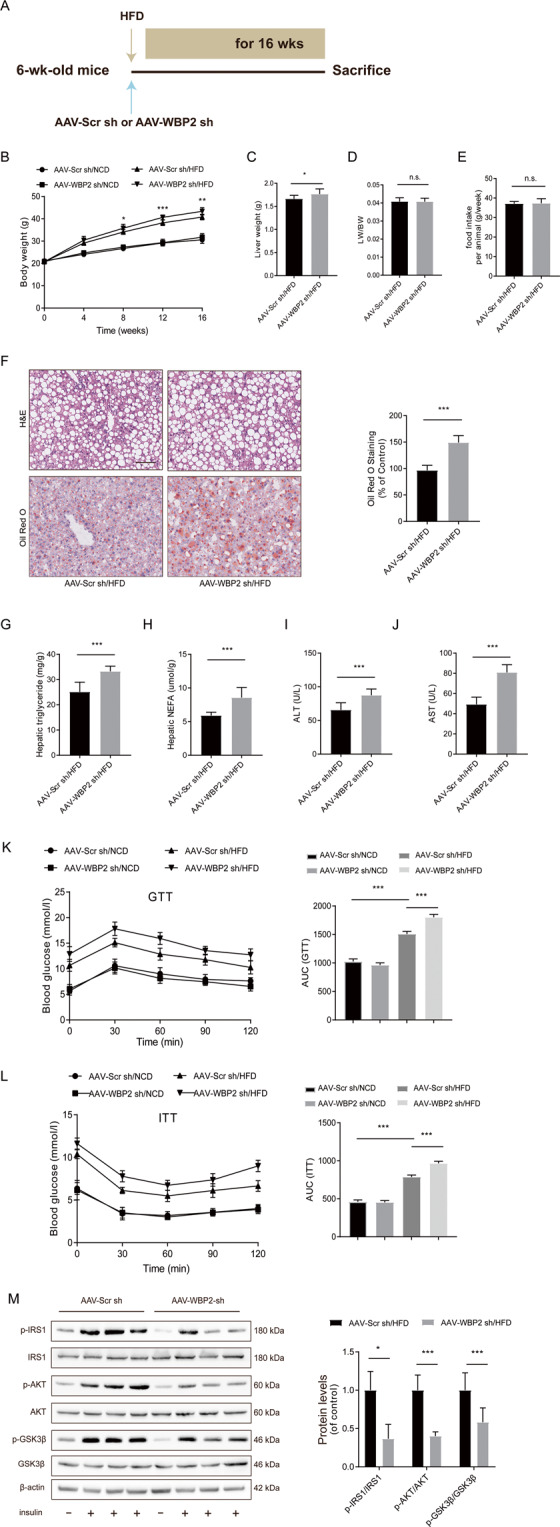
WBP2 knockdown aggravates HFD-induced hepatic steatosis and insulin resistance. **A** Schedule of WBP2 knockdown. Six-week-old mice were injected with AAV-Scr sh (as a control) or AAV-WBP2 sh (knockdown WBP2) via the tail vein. **B** Body weight, **C** liver weight, **D** LW/BW, and **E** food intake of the AAV-Scr sh mice and the AAV-WBP2 sh mice (*n* = 8/group). **F** Representative images of H&E and Oil red O staining of liver tissues from the AAV-Scr sh mice and the AAV-WBP2 sh mice fed an HFD for 16 weeks. Scale bar, 100 μm. **G**, **H** Triglyceride, NEFA, and **I**, **J** ALT and AST levels in the livers of the AAV-Scr sh mice and the AAV-WBP2 sh mice fed an HFD for 16 weeks (*n* = 8/group). **K**, **L** Intraperitoneal glucose tolerance tests (GTTs; 1 g/kg) (**K**) and intraperitoneal insulin tolerance tests (ITTs; 0.75 U/kg) (**L**) were performed on the AAV-Scr sh mice and the AAV-WBP2 sh mice at the 16th week of food administration. The corresponding area under the curve (AUC) of the blood glucose level was calculated (*n* = 8/group). **M** Representative western blot analysis (*n* = 3 western blots for each band) of phosphorylated (p-) and total IRS1, AKT, and GSK3β expression in the livers of the AAV-Scr sh mice and the AAV-WBP2 sh mice fed an HFD for 16 weeks that received insulin treatment (*n* = 2 mice in each group without insulin injection; *n* = 6 mice in each group with insulin injection). Data represent the mean ± SEM, *P < 0.05, **P < 0.01, ***P < 0.001 and n.s. indicates no significance between the two indicated groups.

To further explore the role of WBP2 in fatty liver, we injected mice with AAV-Scramble shRNA and AAV-WBP2 shRNA via the tail vein followed by 16 weeks of HFD (Fig. 4A). We determined the protein expression of WBP2 in the mouse livers at different time points after virus injection and found that AAV-WBP2 shRNA could achieve long-term knockdown (Supplementary Fig. S4A). The immunofluorescence results also indicated that WBP2 was significantly knocked down (Supplementary Fig. S4B). After 16 weeks of HFD feeding, the weight and liver weight of the mice in the AAV-WBP2 shRNA group increased significantly compared with those in the Scr-sh group (Fig. 4B, C), and the liver weight/body weight did not change significantly (Fig. 4D). There was no difference between the Scr-sh and WBP2-sh groups and the normal diet group in food intake (Fig. 4E). H&E and Oil red O staining showed stronger lipid deposition in the livers of the mice in the WBP2-sh group than the other groups (Fig. 4F). In addition, the TG and NEFA levels were higher in the WBP2-sh group (Fig. 4G, H). Liver injury caused by lipid droplet deposition was also stronger in the WBP2-sh group, indicating that its AST and ALT levels were higher than those in the Scr-sh group (Fig. 4I, J). In terms of glucose metabolism and insulin sensitivity, the GTTs and ITTs results showed that WBP2 knockdown reduced glucose tolerance and insulin sensitivity (Fig. 4K, L). For the acute tissue insulin signaling tests, we measured the phosphorylation levels of important molecules in the insulin pathway, and IRS, AKT, GSK3β, and WBP2 knockdown significantly reduced the phosphorylation levels of these molecules (Fig. 4M). The data above demonstrated that WBP2 hepatocyte deficiency could exacerbate HFD-induced fatty liver and IR.

### WBP2 could affect the AMPK pathway by binding to AMPKβ1

**Fig. 5:**
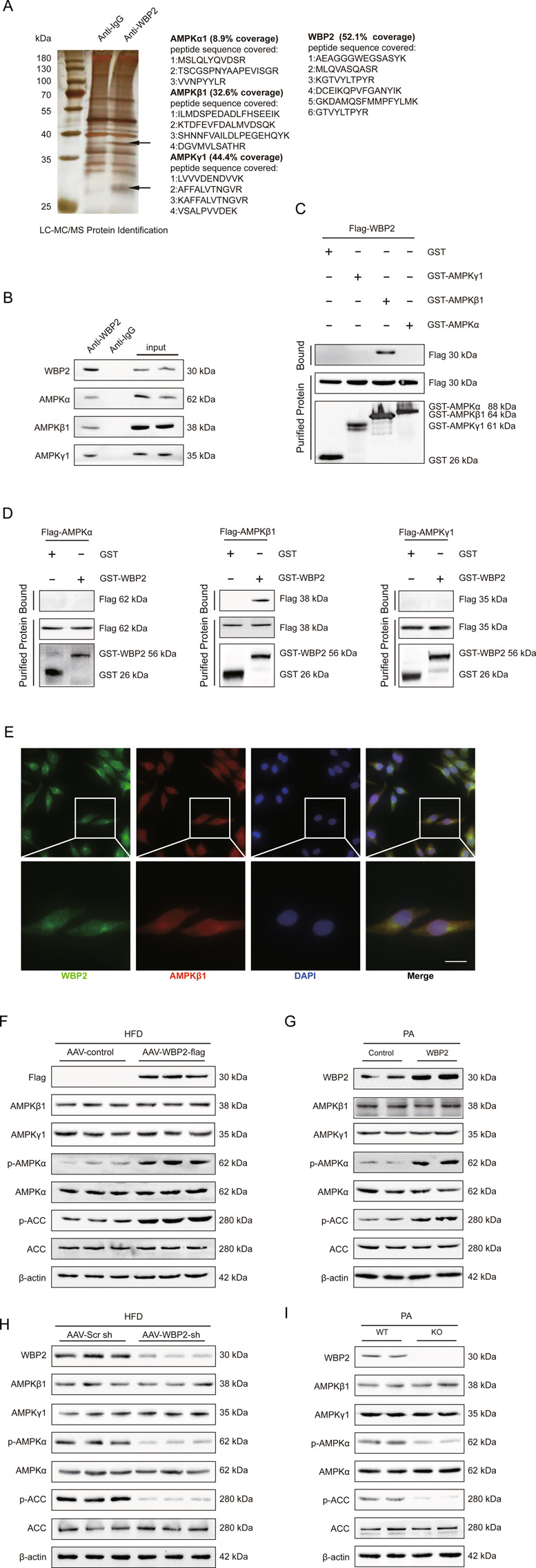
WBP2 could bind to AMPKβ1 and enhance the AMPK pathway in the liver. **A** Silver-stained gel of the indicated proteins binding to WBP2, which were coimmunoprecipitated using an anti-WBP2 antibody and identified via mass spectrometry (MS) in LO2 cells. IgG was used as a control. **B** Immunoprecipitation and western blot assays using anti-WBP2, anti-AMPKα, anti-AMPKβ1, and anti-AMPKγ1 to detect the binding of AMPKα, AMPKβ1, and AMPKγ1 to WBP2 in LO2 cells. IgG was used as a control. **C**, **D** GST precipitation assays using anti-GST and anti-Flag to detect the direct binding of WBP2 and AMPKβ1. Purified GST was used as a control. **E** WBP2 colocalized with AMPKβ1. Immunofluorescence staining for WBP2 (green), AMPKβ1 (red), and DAPI (blue) in LO2 cells. Scale bar, 20 μm. **F** Western blot assays were performed in liver tissues from the AAV-WBP2 mice and the AAV-control mice fed an HFD for 16 weeks to detect the expression of AMPKβ1, AMPKγ1, AMPKα, p-AMPKα, ACC, and p-ACC. Anti-Flag was used to detect the expression of exogenous WBP2, and β-actin served as the loading control. **G** Western blot assays were performed in the PA-treated LO2 cells transfected with WBP2 or control to detect the expression of AMPKβ1, AMPKγ1, AMPKα, p-AMPKα, ACC, and p-ACC. β-actin served as loading control. **H** Western blot assays were performed in liver tissues from the AAV-Scr sh mice and the AAV-WBP2 sh mice fed an HFD for 16 weeks to detect the expression of AMPKβ1, AMPKγ1, AMPKα, p-AMPKα, ACC, and p-ACC. β-actin served as the loading control. **I** Western blot assays were performed in the PA-treated wild-type (WT) or WBP2-knockout (KO) LO2 cells to detect the expression of AMPKβ1, AMPKγ1, AMPKα, p-AMPKα, ACC, and p-ACC. β-actin served as loading control.

To further study the mechanism underlying the protective effect of WBP2 on NAFLD, we used liquid chromatography-tandem mass spectrometry (LC-MS/MS) to screen the interacting proteins. The whole protein sample extracted from LO2 cells was incubated with an anti-WBP2 antibody overnight, and the eluent was electrophoresed and silver-stained the next day. Finally, the different bands were cut out and subjected to LC-MS/MS. Whole protein incubated with an anti-IgG antibody served as a negative control. We found that the AMPK complex was a candidate WBP2-binding protein (Fig. 5A). To further verify our findings, we performed a co-IP experiment. We observed that endogenous AMPKα, β1, and γ1 could coimmunoprecipitate with WBP2 in primary hepatocytes (Fig. 5B). Since the IP experiment cannot determine whether proteins bind directly or indirectly, we used GST precipitation. GST-tagged WBP2 efficiently pulled down AMPKβ1 instead of α or γ1 and vice versa (Fig. 5C, D).

The immunofluorescence colocalization results of WBP2 and AMPKβ1 showed that the two molecules were expressed in the nucleus and cytoplasm, with slightly more in the cytoplasm, and the fluorescence results showed that the two molecules were located together (Fig. 5E).

The above results suggest that WBP2 could combine with the AMPK complex and directly interact with the β1 subunit. WBP2 acts on AMPKβ1, which is a component of the AMPK complex. Considering the important role of AMPK in various metabolic diseases^10,23^, whether the protective effect of WBP2 on the fatty liver is mediated through the AMPK pathway remains unclear. To clarify this issue, we detected changes in related molecules in the AMPK pathway. The phosphorylation of AMPKα and ACC was upregulated in liver samples from the AAV-WBP2 group after consuming an HFD for 16 weeks (Fig. 5F). Then, we transfected the LO2 cell line with WBP2 and stimulated it with PA. The results illustrated that WBP2 overexpression could increase the phosphorylation of AMPKα and ACC (Fig. 5G). Moreover, we demonstrated decreased AMPK pathway activity in the livers of the HFD-fed mice with AAV-WBP2 sh and the LO2 cells with WBP2 knockdown (Fig. 5H, I).

### WBP2 further activated the AMPK pathway by promoting the phosphorylation of AMPKβ1 at Ser108

**Fig. 6:**
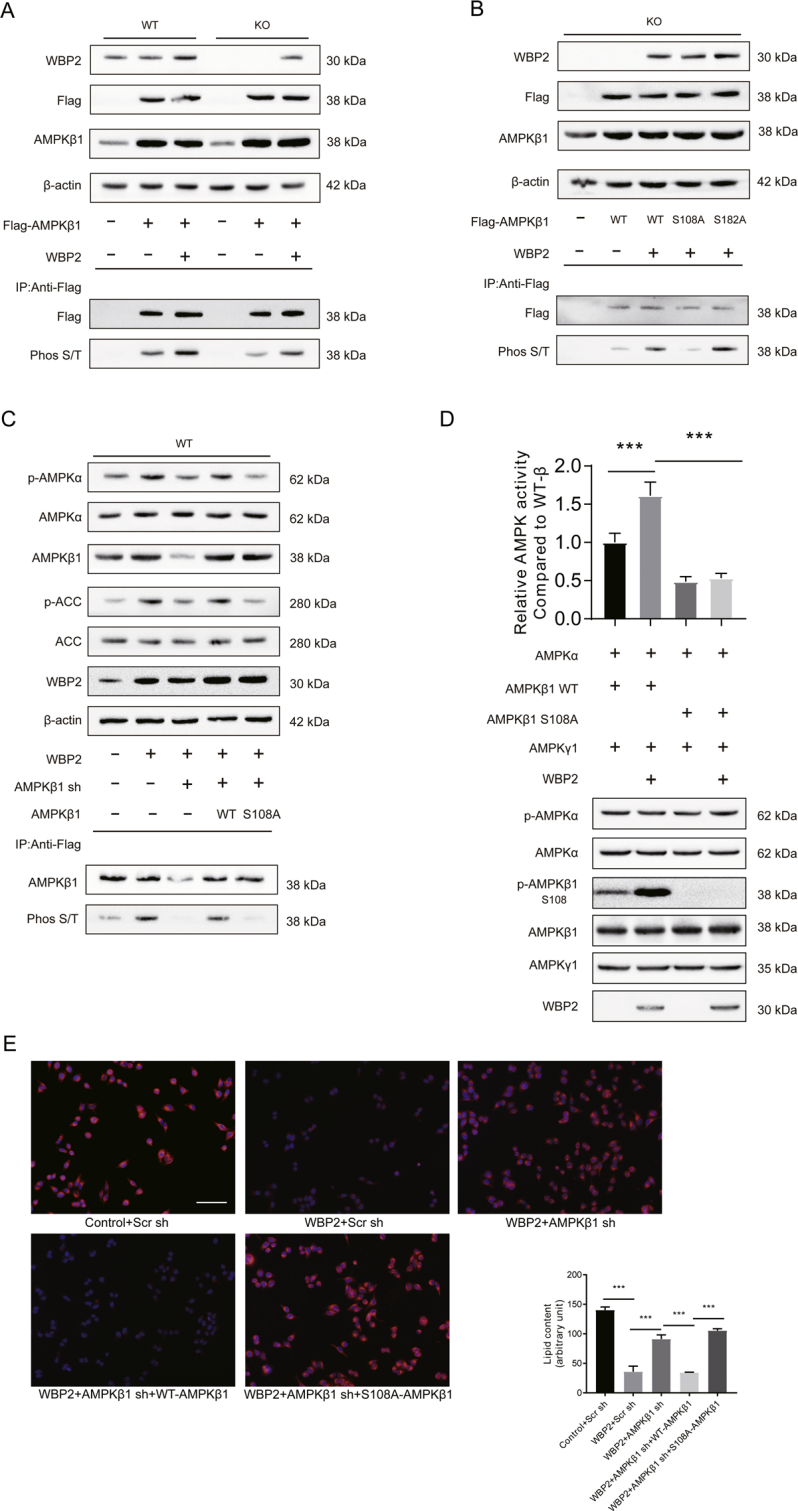
The impact of WBP2 on AMPK is mediated by the phosphorylation of AMPKβ1 at Ser108. **A** Western blot assays using anti-WBP2 and anti-Flag to detect the expression of WBP2 and exogenous AMPKβ1 in WBP2 wild-type (WT) and WBP2 knockout (KO) LO2 cells. β-actin served as loading control. Immunoprecipitation assays using anti-Flag and western blot assays using anti-phospho-(Ser/Thr) (Phos S/T) to detect the phosphorylation of AMPKβ1. **B** Western blot assays using anti-WBP2 and anti-Flag to detect the expression of WBP2 and exogenous AMPKβ1 (including wild-type (WT) or Ser108 mutated to Ala108 (S108A) plasmids) in the WBP2 knockout (KO) LO2 cells. β-actin served as the loading control. Immunoprecipitation assays using anti-Flag and western blot assays using anti-phospho-(Ser/Thr) (Phos S/T) were used to detect the phosphorylation of AMPKβ1. **C** Western blot assays were performed to detect the expression of AMPKα, p-AMPKα, ACC, and p-ACC in the WBP2 WT cells with or without WBP2 overexpression, with or without AMPKβ1 siRNA, and with or without AMPKβ1 WT or with the Ser108 to Ala108 mutation (S108A); anti-WBP2 was used to detect the expression of WBP2, and β-actin served as the loading control. Immunoprecipitation assays using anti-Flag and western blot assays using anti-phospho-(Ser/Thr) (Phos S/T) to detect the phosphorylation of AMPKβ1. **D** Relative AMPK complex activity compared with the wild-type AMPK complex was measured by the ADP-Glo^TM^ kit according to the generation of ADP. Purified AMPK subunits and WBP2 were incubated. Part of the mixture was used to measure luminescence, and western blot assays were used to detect the phosphorylation of AMPKα and AMPKβ. **E** Representative photomicrographs with Nile red staining are shown for the WBP2 WT cells with or without WBP2 overexpression, with or without AMPKβ1 siRNA, and with or without AMPKβ1 WT or S108A exposed to PA (0.25 mM) for 12 h. The Nile red-stained area was quantified by ImageJ software. Scale bar, 100 μm. Data are expressed as the mean ± SEM, ***P < 0.001 between the two indicated groups.

The effect of these three subunits on AMPK complex activity has long been studied^10,24–27^. AMPKβ1 is well known as a scaffold unit, but increasing evidence has elucidated the regulation of the AMPK complex phosphorylation status and activity^28,29^. Multiple sites of AMPKβ1 can be phosphorylated; the phosphorylation of the Ser182 site is related to the nuclear transport of AMPKβ1 itself, whereas phosphorylation of AMPKβ1 at Ser108 could increase AMPK activity^28^. Here, we wanted to explore whether WBP increased AMPK pathway activity by promoting AMKPβ1 phosphorylation. First, we observed that overexpression of WBP2 strongly increased total AMPKβ1 phosphorylation. The total phosphorylated AMPKβ1, which was immunoprecipitated by the anti-Flag antibody, was decreased in the WBP2 KO cell line (Fig. 6A). Next, WT AMPKβ1 and AMPKβ1 with a Ser108 to Ala108 (S108A) mutation or Ser182 to Ala182 (S182A) mutation was separately co-transfected with WBP2 in WBP2 KO cell lines for co-IP experiments.

We found that AMPKβ1 phosphorylation in the S108A mutant group was weaker than that in the WT or S182A mutant group (Fig. 6B). Moreover, knockdown of AMPKβ1 by using siRNA targeting the 3’-UTR of AMPKβ1 reduced WBP2-mediated AMPKβ1 phosphorylation, which could be restored by supplementation of the WT AMPKβ1 instead of the Ser108 mutant (Fig. 6C). WBP2 further activated the AMPK pathway through the phosphorylation of AMPKβ1 at Ser108.

However, Scott et al. found that the phosphorylation of the S108 site of AMPKβ1 could indeed enhance the activity of the AMPK complex in a cell-free test, but it could not enhance the phosphorylation of AMPKα at Thr172^12^. To study the effect of WBP2 on AMPK activity and phosphorylation of Thr172 in AMPKα, we designed a cell-free test as follows: purified AMPK complexes containing WT or S108A β1 subunits were incubated with or without WBP2, and then, the ADP-GLO kit from Promega was used to detect the rate at which the AMPK complex consumes ATP to measure the relative activity of the AMPK complex. Western blotting was applied to detect the phosphorylation of AMPKα and AMPKβ1. The results showed that WBP2 could increase the phosphorylation of S108 of the β1 subunit in a cell-free environment but had no effect on the phosphorylation of the Thr172 site of the AMPKα subunit (Fig. 6D). The difference between the cell-free and cellular tests indicated that the effect of WBP2 on AMPKα phosphorylation might be based on an unknown mechanism, and the effect of WBP2 on the activity of the AMPK complex was based on AMPKβ1. The mechanism of the effect of WBP2 on the phosphorylation of AMPKα requires further research.

To determine the effect of the S108 site of the β1 subunit on lipid deposition in vitro, we overexpressed WBP2 in LO2 cells and then knocked down AMPKβ1 to observe the effect of the wild type and mutant type on lipid droplet deposition. Nile red staining results showed that the effect of WBP2 on lipid droplet deposition in liver cells was dependent on AMPKβ1 and was related to its S108 locus (Fig. 6E). These results showed that WBP2 could enhance the AMPK pathway by promoting the phosphorylation of AMPKβ1 at Ser108.

### AMPKβ1 overexpression alleviated the WBP2 knockdown-induced exacerbation of the fatty liver disease

**Fig. 7:**
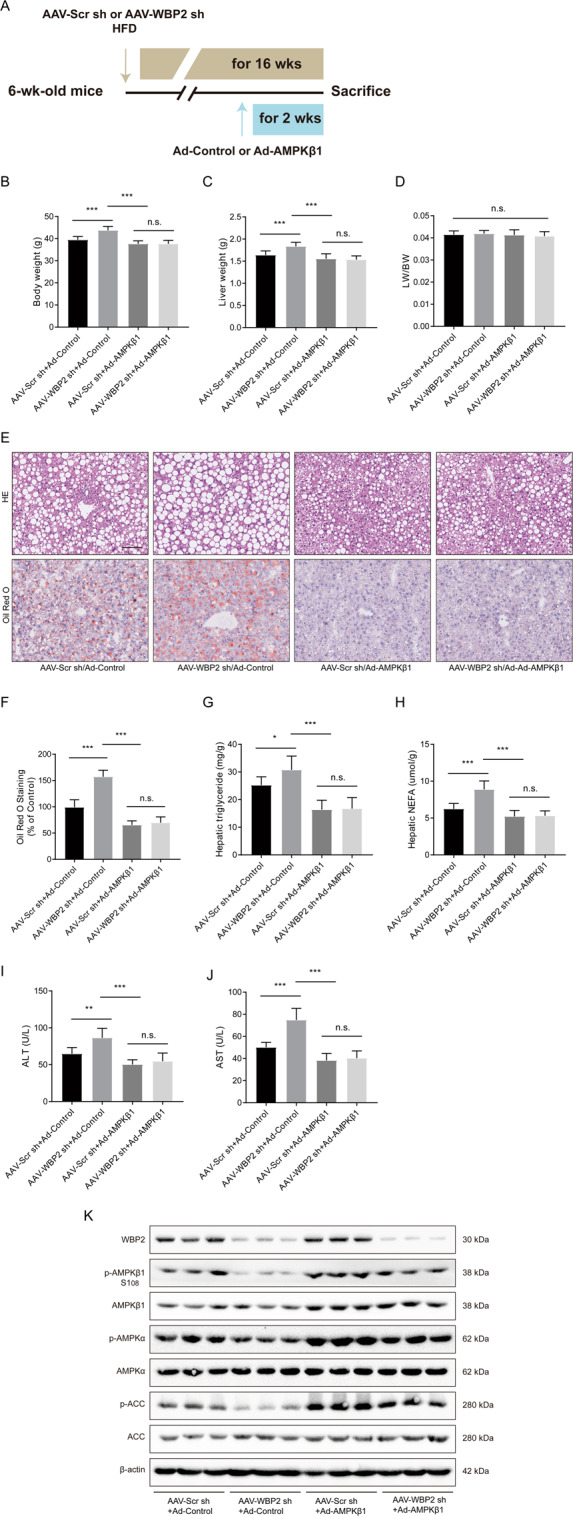
AMPKβ1 reversed the phenotype induced by WBP2 knockdown. **A** Schedule of manipulating the expression of AMPKβ1. Six-week-old mice were injected with AAV-Scr sh (as a control) or AAV-WBP2 sh (knockdown WBP2) via the tail vein and fed an HFD. Ad-Control or Ad-AMPKβ1 was injected at the 14th week after HFD feeding, and the mice above were sacrificed 2 weeks later. **B** Body weight, **C** liver weight, and **D** LW/BW of the mice from the indicated groups (*n* = 8/group). **E**, **F** Representative images of H&E and Oil red O staining of liver tissues from the mice from the indicated groups. Scale bar, 100 μm. **G**, **H** Triglyceride, NEFA, and **I**, **J** ALT and AST levels in the livers of the mice from the indicated groups fed an HFD for 16 weeks (*n* = 8/group). **K** Western blot assays were performed to detect the expression of WBP2, p-AMPKβ1, AMPKβ1, p-AMPKα, AMPKα, p-ACC, and ACC in the livers of the mice from the indicated groups. β-actin served as the loading control. Data represent the mean ± SEM, *P < 0.05, **P < 0.01, ***P < 0.001 and n.s. indicates no significance between the two indicated groups.

To explore whether AMPKβ1 could reverse the phenotype induced by WBP2 knockdown, we fed mice that had been injected with AAV-Scr sh or AAV-WBP2 an HFD for 14 weeks and then injected Ad-AMPKβ1 into the tail vein. After continued HFD feeding for 2 weeks, the phenotype was tested (Fig. 7A). The body weight and liver weight of the mice in the Ad-AMPKβ1 group were significantly lower than those in the Ad-control group (Fig. 7B, C), and there were no significant changes in the liver/body weight ratio (Fig. 7D) or food intake (data not shown). H&E and Oil red O staining of mouse livers showed that overexpression of AMPKβ1 alleviated lipid deposition in the liver caused by an HFD (Fig. 7E, F). Liver injury indexes caused by fatty liver, AST, ALT, and liver TG and NEFA levels (Fig. 7G–J) were all decreased in the AMPKβ1 overexpression group. We examined the protein expression level of WBP2 and its downstream molecules in mouse livers and found that the overexpression of AMPKβ1 could enhance the phosphorylation of the AMPK pathway (including AMPKα and ACC) caused by WBP2 knockdown (Fig. 7K). Thus, WBP2 influenced the AMPK pathway through AMPKβ1.

### PA affected WBP2 expression through miR-27a-5p

**Fig. 8:**
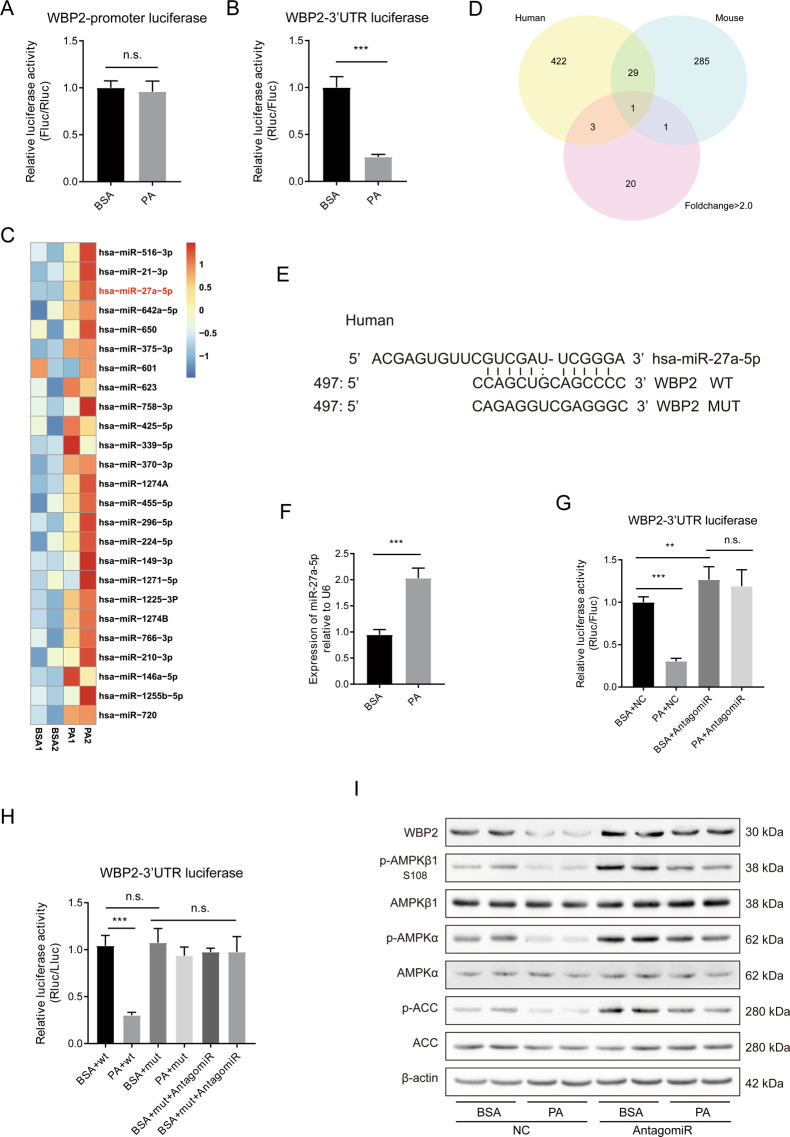
PA influenced WBP2 expression by upregulating miR-27a-5p. **A** LO2 cells were transfected with the WBP2 promoter reporter plasmid and treated with BSA or PA. The luciferase activity was analyzed (*n* = 5 per group). **B** The WBP2-3′-UTR reporter plasmid was transiently transfected into LO2 cells and stimulated as indicated. The luciferase reporter assay was analyzed (*n* = 5 per group). **C** Heat maps showing the changes in the expression of DEGs involved in microRNA upregulation by PA treatment. The color bar shows the gradient of the log2-fold changes in microRNA expression levels in the PA-treated samples relative to those in the BSA-treated samples. **D** MicroRNAs targeting the 3’-UTR of WBP2 in humans and mice were predicted using multiple databases. A Venn diagram was constructed with the upregulated miRNAs in the heatmap, the intersection was identified, and miR-27a-5p was discovered. **E** Binding site of miR-27a-5p on the 3’-UTR of WBP2 in human. **F** The relative expression of miR-27a-5p between BSA- and PA-treated LO2 cells. **G** The WBP2-3′-UTR reporter plasmid was transfected into LO2 cells with miR-27a-5p antagomir and stimulated with PA or BSA. The luciferase reporter assay was performed (*n* = 5 per group). **H** Wild-type or mutated WBP2 3’-UTR reporters were transfected into LO2 cells with or without miR-27a-5p and stimulated with PA or BSA. The luciferase reporter assay was performed (*n* = 5 per group). **I** Wild-type or mutated WBP2 3’-UTR reporters were transfected into LO2 cells with or without miR-27a-5p and stimulated with PA or BSA. Western blot assays were performed to detect the expression of WBP2, p-AMPKβ1, AMPKβ1, p-AMPKα, AMPKα, and p-ACC and ACC, and β-actin served as a loading control. Data represent the mean ± SEM, **P < 0.01, ***P < 0.001 and n.s. indicates no significance between the two indicated groups.

We conducted a series of experiments to determine the mechanism by which PA regulates WBP2 expression. We first constructed the promoter reporter gene of WBP2 and found that PA stimulation did not affect the activity of the WBP2 promoter (Fig. 8A), suggesting that PA regulation of WBP2 expression may be at the post-transcriptional level. Given the important role of miRNAs in post-translational modifications, we generated the 3’-UTR of the WBP2 luciferase vector using the psiCHECK-v2 plasmid and found that PA stimulation could reduce the activity of the WBP2 3’-UTR reporter (Fig. 8B). These results suggested that there might be an important microRNA that plays a role in the regulation of WBP2 expression. After a search of the literature, we found a dataset of miRNA changes after PA stimulation in the GSE database^30^. We made a heatmap based on these results (Fig. 8C). In addition, we queried multiple miRNA target gene prediction databases, and by setting the screening adjustments, we found that multiple databases predicted the same site, and both human and mouse WBP2 3’-UTRs could be combined. Finally, miR-27a-5p was selected as a possible candidate (Fig. 8D). We hypothesized that PA stimulation inhibited WBP2 expression by increasing miR-27a-5p. Figure 8E shows the possible binding sites of miR-27a-5p and the 3’-UTR of WBP2, and we constructed a mutant reporter gene. We first examined miR-27a-5p expression after PA stimulation and found that it indeed increased (Fig. 8F). Then, we administered the miR-27a-5p antagonist with or without PA stimulation to detect the activity of the WBP2 3’-UTR reporter gene and found that the miR-27a-5p antagonist significantly increased the 3’-UTR activity level (Fig. 8G) but had no effect on the mutant (Fig. 8H). This finding suggests that miR-27a-5p inhibits WBP2 expression by binding to the predicted sites of the WBP2 3’-UTR. Moreover, we tested the effect of miR-27a-5p antagonists on the WBP2 protein levels and found that the miR-27a-5p antagonist could increase the WBP2 protein expression, and WB showed that the miRNA antagonist could also increase AMPKα, p-AMPKα, AMPKβ1, p-AMPKβ1, ACC, and p-ACC (Fig. 8I). These results suggest that PA can inhibit WBP2 protein expression by increasing miR-27a-5p.

## Discussion

With the development of society, the incidence of metabolic diseases is increasing, and the identification of new drug targets is urgently needed. To date, there is no specific drug for NAFLD on the market^31^. For people suffering from metabolic diseases such as NAFLD or obesity, doctors generally recommend increased exercise, dietary changes, and maintenance of a healthy lifestyle. Finding specific target molecules for abnormal lipid metabolism is a hot spot in research on NAFLD.

Our study found that the expression of WBP2 decreased in the fatty liver due to a HFD, and overexpression of WBP2 reduced fatty liver and IR. Moreover, we confirmed that WBP2 activated the AMPK pathway by interacting with AMPKβ1 and phosphorylating it at Ser108. These results showed that WBP2 might become a gene therapy target for fatty liver. One limitation of this study is that although we found that WBP2 can increase the phosphorylation of AMPKα and increase the activity of AMPK, the exact mechanism by which WBP2 affects the phosphorylation of the α subunit is not clear. This issue requires further research.

The AMPK agonist metformin, as a first-line medication for diabetes^32–34^, has a significant effect on reducing fatty liver lipid deposition caused by HFD in preclinical rodent experiments^35–37^. However, the results of clinical trials showed that metformin has little effect on fatty liver^38–41^. The reason for the difference between the mouse test and the human test may be related to the concentration and timing of the metformin treatment in clinical tests or the existing species differences, and there may be other unknown reasons. Does the poor effect of metformin on human NFALD mean that the therapeutic effect of activating the AMPK pathway on NFALD in human trials is unsatisfactory? As many scholars have proven the important role of AMPK in metabolic diseases^9,13,26,27^, it is still necessary to find new therapeutic targets for the AMPK pathway. Changes in the energy state of the cell and an increase in the ratio of AMP/ATP can activate AMPK, and metformin activates AMPK by changing the ratio of AMP/ATP^5,42^. There are also some small-molecule agonists that activate AMPK through a different mechanism of action than metformin. For example, direct AMPK activators can activate AMPK independently of changes in cellular energy status. Synthetic direct activators have been designed to interact with the β subunit of AMPK specifically. These AMPKβ-specific activators include A-769662^43^, 991^44^, and MT 63-78^45^. The study of A-769662 in NAFLD shows that in mouse models, A-769662 could reduce liver malonyl-CoA levels, increase fatty acid oxidation, and reduce liver TG in murine models^43,46^. Targeting AMPK by direct agonists, such as small-molecule drug A-769662, may be a new idea for the future design of NAFLD targets. This study clarified the effect of WBP2 on the phosphorylation of the AMPKβ1 S108 site, which may be a new target for future drug design. Of course, the effect of WBP2 on the fatty liver may not depend on the AMPK mechanism, and we need to explore this issue in the future further.

In summary, the current work demonstrates the relationship between the WBP2 and AMPK pathways in regulating lipid metabolism and NAFLD progression. These findings suggest that gene therapy, such as recombinant AAV-WBP2 therapy, may be a promising strategy for treating hepatic steatosis and related metabolic dysfunctions.

## Supplementary information

Supplementary Figure legend

Figure S1

Figure S2

Figure S3

Figure S4
