# Supplementary material for: WW domain-binding protein 2 overexpression prevents diet-induced liver steatosis and insulin resistance through AMPKβ1
Source: Cell Death Dis. 2021 Mar 3;12(3):228. doi: 10.1038/s41419-021-03536-8 (PMC7930037; doi:10.1038/s41419-021-03536-8)
Supplement: Supplementary file 1 — Supplementary Figure legend [file 41419_2021_3536_MOESM1_ESM.docx]

**Supplementary Figure legend**

**Fig. S1.**

(A, B) The normal human hepatocyte cell line LO2 was stimulated with OA for 24 h, and WBP2 expression was examined by Western blots (A) and RT-PCR. (B) (n = 3 independent experiments). Quantification of protein and mRNA expression levels with normalization to β-actin levels. Data are expressed as the mean ± SEM, n.s. indicates no significance between the two indicated groups.

**Fig. S2.**

(A) Western blot assays using anti-WBP2 to detect the expression level of WBP2 in primary hepatocytes isolated from adult mice and infected with adenovirus containing GFP, WBP2. (B) Representative photomicrographs with Nile red staining are shown for GFP and WBP2 cells exposed to PA (0.25 mM) for 12 h. (C) Western blot assays using anti-WBP2 to detect the expression level of WBP2 in primary hepatocytes isolated from adult mice and infected with adenovirus containing scramble shRNA, and WBP2 shRNA. (D) The mRNA expression of WBP2 was tested by RT-PCR analysis. (E) Representative photomicrographs with Nile red staining are shown for scramble shRNA and WBP2 shRNA cells exposed to PA (0.25 mM) for 12 h. The Nile red-stained area was quantified by ImageJ software. Scale bar, 100 μm. Data are expressed as the mean ± SEM, ****P* < 0.001.

**Fig. S3**

(A) Western blot assays using anti-WBP2 to detect the expression level of WBP2 in the livers of the mice injected with AAV-Control and AAV-WBP2 fed an HFD for different times. (B) Western blot assays were performed using an anti-Flag antibody to detect the expression of exogenous WBP2 in the different organs of mice fed an HFD for 16 weeks. (C) Representative images of immunofluorescence staining for WBP2 (green), ALB (red) and DAPI (blue) in liver samples from the AAV-Control mice and the AAV-WBP2 mice fed an HFD for 16 weeks. Scale bar, 100 μm (n =8/group).

**Fig. S4**

(A) Western blot assays using anti-WBP2 to detect the expression level of WBP2 in the livers of the mice injected with AAV-Scr sh and AAV-WBP2 sh fed an HFD for different times. (B) Representative images of immunofluorescence staining for WBP2 (green), ALB (red) and DAPI (blue) in liver samples from the AAV-Scr sh mice and the AAV-WBP2 sh mice fed an HFD for 16 weeks. (n =8/group). Scale bar, 100 μm.
