# Supplementary figures and images for: WW domain-binding protein 2 overexpression prevents diet-induced liver steatosis and insulin resistance through AMPKβ1
Source: Cell Death Dis. 2021 Mar 3;12(3):228. doi: 10.1038/s41419-021-03536-8 (PMC7930037; doi:10.1038/s41419-021-03536-8)

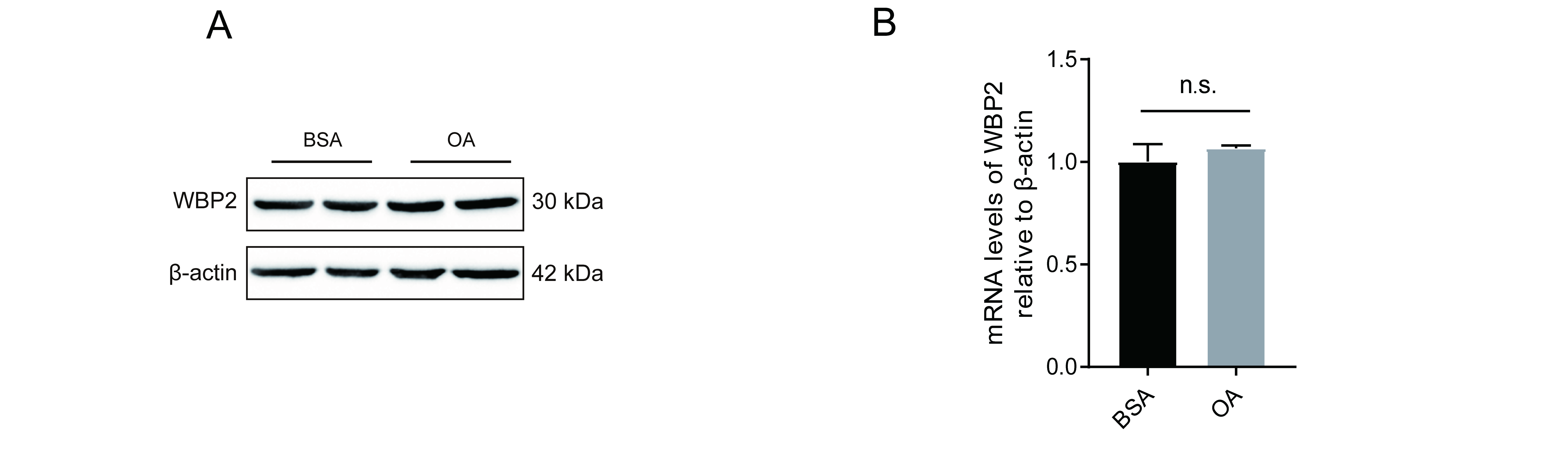

Supplement: Supplementary file 2 — Figure S1 [file 41419_2021_3536_MOESM2_ESM.tif]
